# Differential expression and regulation of *ADAD1*, *DMRTC2*, *PRSS54*, *SYCE1*, *SYCP1*, *TEX101*, *TEX48*, and *TMPRSS12* gene profiles in colon cancer tissues and their *in vitro* response to epigenetic drugs

**DOI:** 10.1371/journal.pone.0307724

**Published:** 2024-08-29

**Authors:** Mikhlid H. Almutairi, Turki M. Alrubie, Alaa T. Alshareeda, Nada Albarakati, Alhomidi Almotiri, Abdullah M. Alamri, Bader O. Almutairi, Mohammad Alanazi

**Affiliations:** 1 Zoology Department, College of Science, King Saud University, Riyadh, Saudi Arabia; 2 Laboratories Directorate, General Directorate of Animal Health, Ministry Deputyship for Agriculture, Ministry of Environment, Water and Agriculture, Riyadh, Saudi Arabia; 3 Blood and Cancer Research Department, King Abdullah International Medical Research Center, King Saud bin Abdulaziz University for Health Sciences, Ministry of National Guard Health Affairs, Riyadh, Saudi Arabia; 4 Blood and Cancer Research Department, King Abdullah International Medical Research Center, King Saud bin Abdulaziz University for Health Sciences, Ministry of National Guard Health Affairs, Jeddah, Saudi Arabia; 5 Department of Clinical Laboratory Sciences, College of Applied Medical Sciences, Shaqra University, Ad Dawadmi, Saudi Arabia; 6 Department of Biochemistry, Genome Research Chair, College of Science, King Saud University, Riyadh, Saudi Arabia; University of the Punjab, PAKISTAN

## Abstract

Colon cancer (CC) is a significant cause of death worldwide, particularly in Saudi Arabia. To increase the accuracy of diagnosis and treatment, it is important to discover new specific biomarkers for CC. The main objectives of this research are to identify potential specific biomarkers for the early diagnosis of CC by analyzing the expressions of eight cancer testis (CT) genes, as well as to analyze how epigenetic mechanisms control the expression of these genes in CC cell lines. Tissue samples were collected from 15 male patients with CC tissues and matched NC tissues for gene expression analysis. The expression levels of specific CT genes, including *ADAD1*, *DMRTC2*, *PRSS54*, *SYCE1*, *SYCP1*, *TEX101*, *TEX48*, and *TMPRSS12*, were assessed using quantitative techniques. To validate the gene expression patterns, we used publicly available CC statistics. To investigate the effect of inhibition of DNA methylation and histone deacetylation on CT gene expression, *in vitro* experiments were performed using HCT116 and Caco-2 cell lines. There was no detected expression of the genes neither in the patient samples nor in NC tissues, except for *TEX48*, which exhibited upregulation in CC samples compared to NC tissues in online datasets. Notably, CT genes showed expression in testis samples. *In vitro*, experiments demonstrated significant enhancement in mRNA expression levels of *ADAD1*, *DMRTC2*, *PRSS54*, *SYCE1*, *SYCP1*, *TEX101*, *TEX48*, and *TMPRSS12* following treatment with 5-aza-2’-deoxycytidine and trichostatin A in HCT116 and Caco-2 cell lines. Epigenetic treatments modify the expression of CT genes, indicating that these genes can potentially be used as biomarkers for CC. The importance of conducting further research to understand and target epigenetic mechanisms to improve CC treatment cannot be overemphasized.

## Introduction

Colon cancer (CC) is a multifaceted and varied disease affected by genetic mutations and epigenetic modifications of proto-oncogenes and key tumor suppressor genes, leading to the growth of colorectal neoplasms. The molecular changes cause the transformation of normal colonic epithelium into invasive and metastatic tumors [1,2]. CC ranks as the third most common in men and fourth in women worldwide in terms of incidence, indicating that it is an important global health problem [3]. According to 2020 statistics, the disease accounts for more than 10.2% (or 1.85 million cases) of all cancer diagnoses and 9.4% (or 850,000 cases) of cancer-related deaths worldwide [4]. The expected doubling of CC prevalence by 2030 is somewhat worrying [5]. Given the current detrimental impact of CC on public health, these statistics highlight the urgent need for more investigation of the disease and improvements in prevention and treatment methods.

In Saudi Arabia, CC is the top and third most common cancer in men and women, respectively [6,7]. It is the leading cause of mortality for both sexes in the Saudi population, accounting for 15.2% of all cancer deaths [8]. Furthermore, early-onset CC is common in Saudis aged >50 years [9], and up to 50% of Saudi patients with CC are diagnosed at advanced stages [10]. However, a recent study found that CC is more prevalent among younger Saudi people [11]. The main challenges faced by the medical community regarding CC are that it is often diagnosed when symptoms emerge at late stages and that the effectiveness of the available treatments is limited [12]. However, early detection of CC can increase the treatment success rate because CC is a slow-progressing disease [12]. Owing to the high mortality rate, widespread prevalence of CC, and the aforementioned challenges, it is vital to find new potential diagnostic and prognostic biomarkers for the early detection of CC to lower its incidence rates [6,7,13].

Potential cancer biomarkers have been studied extensively over the last several decades, focusing on many antigen groups such as the cancer-testis antigen (CTA) class. Antigens in this class could be used as potential biomarkers for the early detection of different types of cancer [6,7,13–15]. In humans, cancer-testis (CT) genes restrictedly encode these CTAs in the germline cells of the testis in normal tissues but may be aberrantly expressed in several types of cancer [13,14]. For example, CTAs are aberrantly expressed in melanoma, ovarian cancer [14], breast cancer [16], leukemia [17], glioblastoma [18], and CC [6,13]. In addition, a previous investigation suggested that in malignancies, CTAs may induce cancer molecular mechanisms such as tumor development, proliferation, and/or antiapoptotic mechanisms [19]. Consequently, the unique restricted expression patterns of CTAs in testicular and cancer cells make them ideal candidate biomarkers for use in early tumor-specific diagnostic and immunotherapeutic approaches owing to their high specificity and lower toxicity than chemotherapy agents [20,21].

Many studies have examined the expressions of CT genes in numerous malignancies and identified potential specific biomarkers for early tumor detection. For example, *MAGE-A1* expression was found in six cancer types, namely colon, lung, cervical, and ovarian cancers, melanoma, and leukemia, but was found only in normal testicular tissues [22]. In addition, Scanlan et al. (2004) reported that selected CT genes were not expressed in renal, pancreatic, and gastric cancers but were highly expressed in bladder, lung, and melanoma malignancies [23]. The gene expressions of *MAGEA1*, *CTAG1A*, *TEX33*, *MAGEB1*, *SPZ1*, *LYZL6*, *SSX1*, *SCP2D1*, *TKTL2*, and *SSX2* were recently found in CC tissue samples from a Saudi population, but not in normal colon (NC) tissues from the same individuals [6,13,24].

DNA methyltransferases (DNMTs) are enzymes that add a methyl group (CH3) to the 5’ position of cytosine bases in CpG (-cytosine-phosphate-guanine-) dinucleotides [25]. Promoter hypermethylation is characterized by increased methylation of the CpG island and often linked to gene silencing. Conversely, promoter hypomethylation is characterized by decreased methylation and can activate the expressions of silenced genes in cancer cells [26]. The development of epigenetic drugs has been driven by the reversible nature of epigenetic alterations, with the aim of testing their efficacy as therapeutic agents against various types of malignancies [27]. The use of epigenetic drugs such as the DNA methyltransferase inhibitor has been shown to enhance CT gene expression in tumors [28], which may lead to the recognition of these genes as non-self by the immune system, hence offering potential applications in the field of cancer immunotherapy [29].

DNA methylation and histone acetylation mechanisms regulate the expressions of numerous CT genes by DNMTs and histone acetyltransferase enzymes (HATs), respectively [7,13,30,31]. HATs are enzymes that transfer the acetyl group from acetyl-CoA to specific lysine residues in histone proteins [32]. Histones are acetylated by HATs, resulting in a dispersed structure of chromatin, which becomes accessible by transcriptional factors. As a result, this this process is crucial for gene regulation in eukaryotic cells [33]. Many CT genes can also be controlled by adding an acetyl group via HATs and by removing an acetyl group via histone deacetylases, resulting in chromatin remodeling via hyperacetylation and hypoacetylation, respectively [30]. Furthermore, the expressions of numerous CT genes can be upregulated after treatment of CC cell lines with a DNA methyltransferase inhibitor (DNMTi), such as 5-aza-2’-deoxycytidine (5-aza-CdR) and histone deacetylase inhibitor (HDACi) [7,11,13,30,31]. Recent studies have reported that treatment with 5-aza-CdR and TSA agents activate the expressions of some CT genes, including *MAGE*-A4, *SSX1*, *SCP2D1*, *MAGE-B1*, *CTAG1A*, *SSX2*, and *SSX3* in CC cell lines. Therefore, these epigenetic modulators (5-aza-CdR and TSA) can be included in the clinical immunotherapy protocol for treating CC owing to their roles in the transcriptional activation of CT genes [7,13].

This research aims to study in depth the expression profiles and regulatory mechanisms of a panel of CT genes in patients with CC. The aim is twofold: first, to discover potential CC-specific biomarkers that can be used for early diagnosis and treatment; and second, understand the epigenetic controls that drive these genes. The CT genes selected for this study were ADAD1, DMRTC2, PRSS54, SYCE1, SYCP1, TEX101, TEX48, and TMPRSS12. These genes have been shown to have testis-restricted expression in humans, according to credible sources such as the GeneBank (https://www.ncbi.nlm.nih.gov/gene (accessed on 4 March 2024)) and CT database (http://www.cta.lncc.br (accessed on 4 March 2024)). To find differential expression patterns that may be diagnostic indicators or prognostic markers of CC, this study aims to conduct an in-depth analysis of the expression profiles of these CT genes using samples from tissues derived from CC patients and adjacent NC tissues. Additionally, CC cell lines were used to perform *in vitro* experiments to study how epigenetic agents, such as histone deacetylase inhibitors and DNA methylation inhibitors, affect the expression levels of selected CT genes. Our goal is to understand how these agents regulate CT gene expression in CC and to find therapeutic targets that can be used to intervene in this process.

## Materials and methods

### Ethical approval and patient sample collections

The institutional review board (reference No. IRB/1201/22; study No. SP22R/076/04) of the King Abdullah International Medical Research Center authorized and approved this study as ethically sound. The study subjects were recruited from King Khalid University Hospital in Riyadh, Saudi Arabia (from 17/07/2022 to 09/07/2023). No treatment, not even chemotherapy or physical therapy, was given to any of the patients. Surgeons and pathologists worked together to confirm the cancer diagnosis using standard clinical, endoscopic, radiological, and histological criteria to identify patients eligible to participate in the research. After reading and understanding the privacy statement, each patient signed a written informed consent form. The participants were then asked to complete a self-administered survey questionnaire on their lifestyle habits, including how often they went out with friends, whether they smoked cigarettes, and how often they drank alcohol. Tissue samples were collected from 15 Saudi men with CC and compared with NC from the same patients. RNA was extracted from the CC and NC tissue samples and stored in individual sterile tubes containing an RNAlater solution (76106; Thermo Fisher Scientific).

### Clinical data of the study subjects

The demographic and health information of the study participants is included in Table 1. Fifteen participants with either NC or CC participated in this trial. The patients with CC had a mean age of 65 years (range 35–96 years) and divided into two broad age groups: those younger than 65 years (47%) and those older than 65 years (53%). Grade 2 CC was found in most patients (80%). Furthermore, Table 1 provides information on the ages, cancer grades, and TNM staging of the patients with CC, among other clinical features.

**Table 1:** Summary of demographic and clinical data of the patients with CC and participants with NC.

Characteristic			CC (n%)			NC (n%)	
No. of patients, n (%)			15 (100)			15 (100)	
Age, mean (range), years			65 (35–96)			65 (35–96)	
<65, n (%)			7 (47%)			7 (47%)	
>65, n (%)			8 (53%)			8 (53%)	
CC patients							
**Patient no.**	**Age, years**	**Cancer grade**		**TNM stage**	**Type of CC**		**Cancer position**
1	49	2		T2N1M0	Invasive adenocarcinoma		Ascending colon
2	69	2		T2N2M0	Invasive adenocarcinoma		Ascending colon
3	65	2		T3N1M0	Invasive adenocarcinoma		Ascending colon
4	63	2		T3N1M0	Invasive adenocarcinoma		Descending colon
5	38	1		T1N0M0	Invasive adenocarcinoma		Descending colon
6	35	1		T1N0M0	Invasive adenocarcinoma		Descending colon
7	69	2		T1N1M0	Invasive adenocarcinoma		Transverse colon
8	96	2		T1N0M0	Invasive adenocarcinoma		Descending colon
9	71	2		T3N0M0	Invasive adenocarcinoma		Ascending colon
10	70	2		T3N2Mx	Invasive adenocarcinoma		Transverse colon
11	56	1		T3N1M0	Invasive adenocarcinoma		Descending colon
12	65	2		T3N0Mx	Invasive adenocarcinoma		Descending colon
13	80	2		T3N0Mx	Invasive adenocarcinoma		Descending colon
14	67	2		T3N1Mx	Invasive adenocarcinoma		Ascending colon
15	83	2		T2N0Mx	Invasive adenocarcinoma		Descending colon

Abbreviations: CC: Colon cancer; NC: Normal colon; TNM: Tumor‑node‑metastasis.

### Sources, cultures, and epigenetic drug treatment (5-Aza-CdR or TSA) of human CC cell lines

Two CC cell lines were tested, HCT116 and Caco-2 cells, which were generously donated by Dr. Bader Almutairi (King Saud University, College of Sciences, Riyadh, Saudi Arabia). They were cultured using Dulbecco’s modified Eagle medium (61965026; Thermo Fisher Scientific) with 10% fetal bovine serum (A3160801; Thermo Fisher Scientific) and stored in a humidified incubator at 37°C with 5% CO_2_ The final 5-aza-CdR and TSA concentrations used in the epigenetic drug treatments were determined after the drugs were dissolved in a DMSO solvent. The cells in each cell line were subcultured according to the following four groups of epigenetic drug treatment: (1) 5-aza-CdR (10 μM for 72 h), (2) DMSO for 72 h (as a negative control), (3) TSA (100 nM for 48 h), and (4) DMSO for 48 h (as a negative control). Our most recent work details how the optimum administration timings and doses of 5-aza-CdR and TSA were established [7,13]. These studies showed that treatment of CC cell lines with 10 μM of 5-aza-2’-deoxycytidine or 100 nM TSA correlated with the activation of X- and non-X-encoded CT genes in somatic cells. In addition, the drugs showed no negative effects on cell viability.

### Isolation of RNA from CC tissues, NC tissues, and cultured cells

Approximately 30 mg of tissue sample for RNA isolation was collected from the patients with CC and the participants with NC. Total RNA was extracted from approximately 5 million cells grown *in vitro*. Then, the All-Prep DNA/RNA Mini Kit (80204; Qiagen) was used to extract total RNA from the CC tissue samples, NC tissue samples, and cultured cells, in accordance with the manufacturer’s guidelines. The isolated RNA concentrations were measured using the methods outlined in previous works [7,13,24].

### Synthesis of complementary DNA

A high-capacity cDNA reverse transcription kit (4368814; Applied Biosystems) was used to convert the total RNA at a concentration of 2000 ng/μL into complementary DNA (cDNA) in accordance with the manufacturer’s instructions. The cDNA was then diluted 1:10 and stored at –20°C, as described in our recent publications [6,7].

### Primers and conditions for RT-PCR and agarose gel electrophoresis

Primers for RT-PCR reactions were developed using a hybrid approach, combining the manual and computational procedures detailed in previous studies [6,7]. All sense and antisense primers were synthesized using Macrogen (Macrogen Inc., Seoul, South Korea). Table 2 lists the sequences of the primers used for the RT-PCR of the chosen genes and the predicted sizes of the PCR products. The cDNA efficacy (quality) was determined by amplifying the housekeeping gene *ACTB* in both the NC and CC groups, and in both the treated CC cell lines. Total RNA from human testicular tissue was utilized for this study (AM7972; Thermo Fisher Scientific) to test the specificity of each gene primer. For the RT-PCR product of each gene, 10.5 μL of nuclease-free water, 1 μL of 200 ng/μL diluted cDNA, 1 μL of 10 μM sense and antisense primers, and 12.5 μL of BioMix Red (BIO-25006; BioLine) were used. Recent papers [3,8,11] detailed the experimental setup, including the heat cycling parameters of the RT-PCR and agarose gel electrophoresis. 8 μL of each PCR product and 2.5 μL of a 100 bp DNA ladder (marker) were separated on a 1.5% agarose gel.

**Table 2:** Sequences of the RT-PCR primers, predicted sizes of the resulting products, and RT-PCR cycling conditions.

Gene symbol	Chr. location	Accession number	Primer category	Primer sequence (5′→3′)	Product size	RT-PCR cycling parameters
*ACTB*	7	NM_001101.5	Sense Antisense	AGAAAATCTGGCACCACACC AGGAAGGAAGGCTGGAAGAG	553 bp	RT-PCR cycling was carried out using the following conditions: one cycle of pre-denaturation at 95°C for 5 min, followed by 35 cycles of denaturation at 95°C for 30s, primer annealing at 58°C for 30s, and extension at 72°C for 30s, and one cycle of final extension at 72°C for 5 min.
*ADAD1*	4	NM_139243.4	Sense Antisense	ATGGCATCCAAGGTTACGCA TCTGGCTTAATGTCCTGGCT	647 bp	
*DMRTC2*	19	NM_001040283.3	Sense Antisense	CAAATGTGTCCTCATCCTGG GCTGGAGTATCAGAGTTGAG	572 bp	
*PRSS54*	16	NM_001080492.2	Sense Antisense	GGAAACCAAGACTGCCTGCT TTGTACAGACGCCTCAGGAG	424 bp	
*SYCE1*	10	NM_001143763.2	Sense Antisense	GAGGTCCTGATTAACCGGAT ATGTCACTGGTCTACCTGGT	787 bp	
*SYCP1*	1	NM_001282541.2	Sense Antisense	TTCAGAGGGATTGAGCAGAG TCAGCTTGCACACGAAGTTC	405 bp	
*TEX101*	19	NM_031451.5	Sense Antisense	TCTGTCCATGACTGTGGAAG CAGTCTTTCGAGGTTGAGTG	569 bp	
*TEX48*	9	NM_001199233.2	Sense Antisense	CCCACCAAAACCTGATCTTG CTGGCAGTAGCGGTTTAAGT	314 bp	
*TMPRSS12*	12	NM_182559.3	Sense Antisense	ACAGCACCGCTTAAGGATGT AGTATGCCTTGAGTGCTTGC	782 bp	

Abbreviations: *ACTB*: Actin beta; *ADAD1*: Adenosine deaminase domain containing 1; *DMRTC2*: Doublesex and mab-3 related transcription factor like family C2; *PRSS54*: Serine protease 54; *SYCE1*: Synaptonemal complex central element protein 1; *SYCP1*: Synaptonemal complex protein 1; *TEX101*: Testis expressed 101; *TEX48*: Testis expressed 48; *TMPRSS12*: Transmembrane serine protease 12; Chr: Chromosome; bp: Base pair.

### qRT-PCR primers, protocol setup, and data analysis

Each pair of qRT-PCR primers was meticulously constructed by hand in accordance with the best practices established in previous studies [6,7]. Primers for qRT-PCR were created, and their sequences and expected sizes are reported in Table 3. The 96-well plate used for the qRT-PCR experiment was iTaq Universal SYBR Green (1725120; Bio-Rad). Each well of the 96-well plate included 10 μL of a mixture consisting 2 μL of 100-ng/μL diluted cDNA, 5 μL of SYBR Green, 0.5 μL of 10 μM sense and antisense primers, and 2.5 μL of nuclease-free water. The qRT-PCR experiment was run three times (triplicate) with a melt-curve analysis, and *GAPDH* expression levels were used as references for normalizing the qRT-PCR data. The qRT-PCR data were analyzed using the SPSS statistical software (version 22; SPSS Inc., Chicago, IL). The following *p* values indicated statistical significance: *p ≤ 0.05, **p ≤ 0.01, ***p ≤ 0.001, and ****p ≤ 0.0001.

**Table 3:** Sequences of the qRT-PCR primers, predicted sizes of the resulting products, and qRT-PCR cycling conditions.

Gene symbol	Primer category	Primer sequence (5′→3′)	Product size	qRT-PCR cycling parameters
*GAPDH*	Sense Antisense	GGGAAGCTTGTCATCAATGG GAGATGATGACCCTTTTGGC	173 bp	Quantitative RT-PCR amplification was performed using the QuantStudioTM 7 Flex Real-Time PCR System and the following protocol: primary denaturation at 95°C for 4 min, followed by 40 cycles at 95°C for 15 s, 58°C for 1 min, and 72°C for 30 s.
*ADAD1*	Sense Antisense	ATGGCATCCAAGGTTACGCA AAACTGGTGCAAGGCTGACA	168 bp	
*DMRTC2*	Sense Antisense	CAAATGTGTCCTCATCCTGG TCTGAAGTGGTTGGGAGCTT	136 bp	
*PRSS54*	Sense Antisense	GGAAACCAAGACTGCCTGCT GCTGTAGTCTTCCACCTTGG	151 bp	
*SYCE1*	Sense Antisense	GAGGTCCTGATTAACCGGAT CTCAAGATCTCCTTCAGGTG	149 bp	
*SYCP1*	Sense Antisense	TTCAGAGGGATTGAGCAGAG CCTGAATGGCTTTTCGCTGT	155 bp	
*TEX101*	Sense Antisense	TCTGTCCATGACTGTGGAAG GCCAAAATGGCTGTCTCAGT	138 bp	
*TEX48*	Sense Antisense	CCCACCAAAACCTGATCTTG TTTTGGGTCGATGGCTTGTG	121 bp	
*TMPRSS12*	Sense Antisense	ACAGCACCGCTTAAGGATGT TAATCTGCAGGCTCACCAC	98 bp	

Abbreviations: *GAPDH*: Glyceraldehyde-3-phosphate dehydrogenase; bp: Base pair.

### Validation of the study cohorts and data analysis

In this study, 308 CC and 41 NC tissue samples from the TCGA colon adenocarcinoma (COAD) dataset were used as the first validation cohort (Cohort 1) for the gene expression analysis. RNA-seq data were examined using OncoDB, a publicly available interactive online portal (https://oncodb.org/) [34]. Cohort 2 was from the GSE32323 (Platform GPL570) dataset from the Gene Expression Omnibus (GEO) database (https://www.ncbi.nlm.nih.gov/geo). It consisted of 17 CC and 17 matched NC tissue samples analyzed using Affymetrix HG-U133 Plus 2.0 arrays. The Student *t* test was performed for the differential expression analysis of the *ADAD1*, *DMRTC2*, *PRSS54*, *SYCE1*, *SYCP1*, *TEX101*, *TEX48*, and *TMPRSS12* genes between the CC and NC tissue samples using GraphPad Prism version 9.5.0 (USA). All differences were considered statistically significant at *p* ≤ 0.05.

## Results

### Evaluation of the expression levels of the selected CT genes in CC and adjacent NC tissues

**Fig 1:**
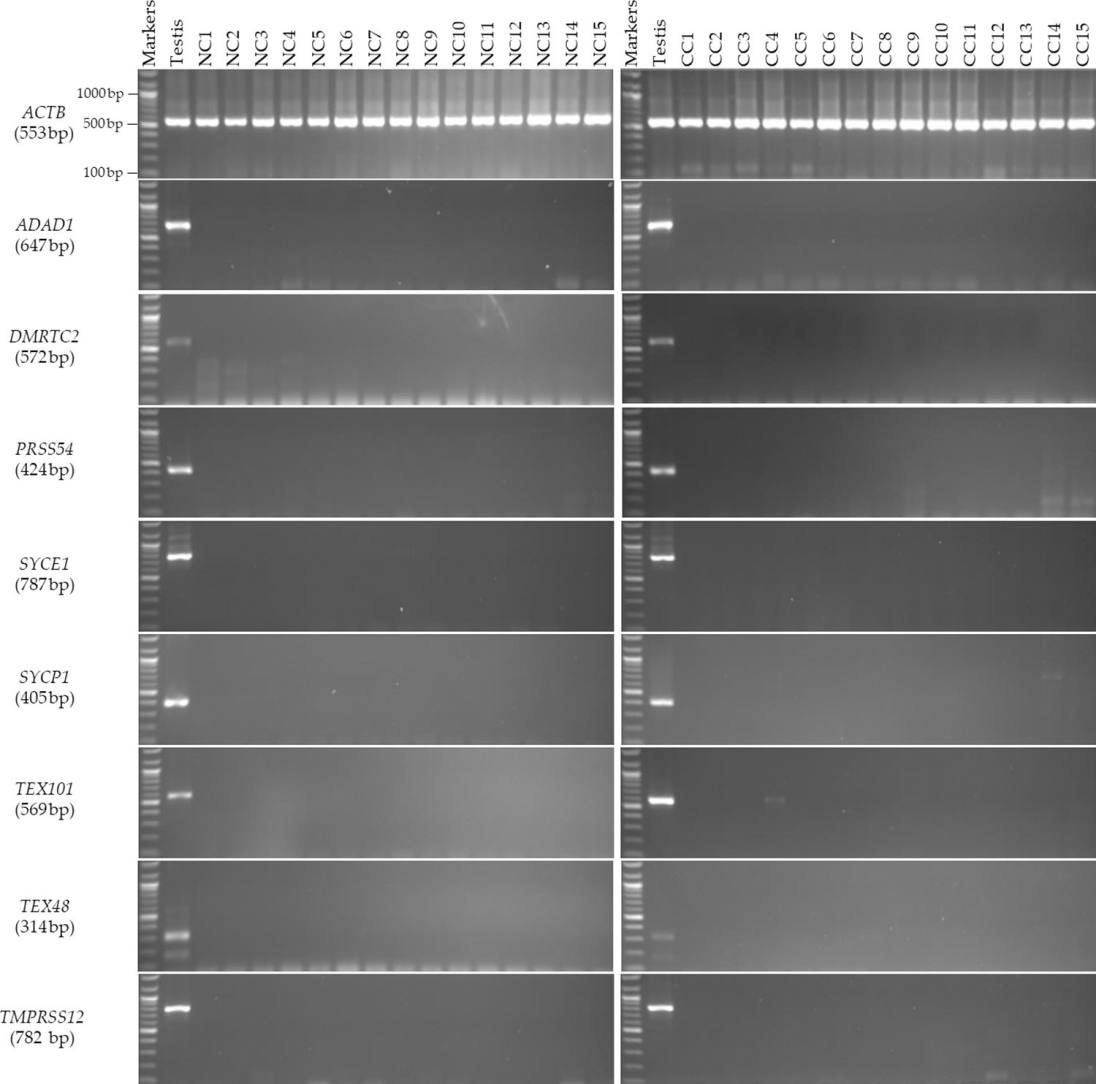
RT-PCR evaluation of the expression patterns of a subset of CT genes in the NC and CC tissue samples from Saudi patients. The results of the RT-PCR analysis of *ADAD1*, *DMRTC2*, *PRSS54*, *SYCE1*, *SYCP1*, *TEX101*, *TEX48*, and *TMPRSS12* are displayed in the agarose gel images. Each cDNA sample was obtained by first isolating total RNA from 15 CC tissue samples and 15 neighboring NC tissue samples. Human *ACTB* expression was used to evaluate the efficacy of each cDNA sample. Human testis cDNA was used to assess the primer quality of each gene. The official symbol and anticipated product size of each studied gene are displayed on the right side of the agarose figure.

In this research, the expressions of eight CT genes, namely *ADAD1*, *DMRTC2*, *PRSS54*, *SYCE1*, *SYCP1*, *TEX101*, *TEX48*, and *TMPRSS12*, were investigated in 15 CC tissue samples and 15 adjacent NC tissue samples from the same Saudi patients to identify genes that could be used as biomarkers for the diagnosis of CC in its early stages. The findings from the reverse transcription polymerase chain reaction (RT-PCR) indicated that the *ADAD1*, *DMRTC2*, *PRSS54*, *SYCE1*, *SYCP1*, *TEX101*, *TEX48*, and *TMPRSS12* genes were not expressed in both the CC and NC tissue samples but were expressed restrictedly in the testis (Fig 1). Thus, the eight CT genes are testis restricted and cannot be considered candidate CC biomarkers in this investigation. However, they may show positive expressions in other CC tissues and cancer tissues from other tumors.

### Sanger sequencing based verification of designed RT-PCR primers in inspected tissue samples

The RT-PCR products of the targeted genes were isolated, sequenced, and compared with GenBank reference sequences using the BLAST program (https://blast.ncbi.nlm.nih.gov/Blast.cgi) to assess the query sequences with those found in NCBI databases. The sequencing of the RT-PCR product was performed to validate the specificity of our results and confirm whether the observed bands corresponded to the targeted genes. As shown in Fig 1, the expected band size for the *SYCP1* gene in the testis was approximately 405 bp based on its primer design (Table 4). However, the RT-PCR product of the CC14 samples showed a larger band size of approximately 700 bp. Thus, the PCR amplicon of this sample was sequenced, and the results of the analyzed sequencing demonstrated that the unexpected large band size was not related to *SYCP1* (Table 4). For the *TEX101* gene, a strange faint band was observed in a CC4 sample at a band size of approximately 620 bp (Fig 1), whereas the anticipated RT-PCR product size was approximately 569 bp (Table 4). However, the DNA sequence result of this strange faint band confirmed no significant sequence similarity to the available sequences of the *TEX101* gene in the GenBank database (Table 4). In addition, only one large faint band of approximately 1000 bp was observed in the *TMPRSS12* RT-PCR products in CC sample 14 (Fig 1), while the *TMPRSS12* primers were designed to produce expected bands of approximately 782 bp in size, as shown in the testis samples where this gene was expressed (Fig 1). Therefore, the CC14 RT-PCR product was sequenced, and the sequencing results indicated that the large faint band was unrelated to the *TMPRSS12* gene (Table 4). Thus, the DNA sequencing results evidenced by the RT-PCR results showed that all the selected CT genes were restricted in the testis and disqualified as CC biomarkers in the Saudi patients.

**Table 4:** Summary of the sequence analysis findings for the *SYCP1*, *TEX101*, and *TMPRSS12* genes in the CC tissue samples.

Gene	Primer direction	Expected RT-PCR product size (bp)	Sequence in CC tissue samples	Sequence identity (%)
***SYCP1***	Sense Antisense	405	14 14	0.00 0.00
***TEX101***	Sense Antisense	569	4 4	0.00 0.00
***TMPRSS12***	Sense Antisense	782	14 14	0.00 0.00

### *In silico* analysis of CT specific gene differential expression between NC and CC tissue samples

**Fig 2:**
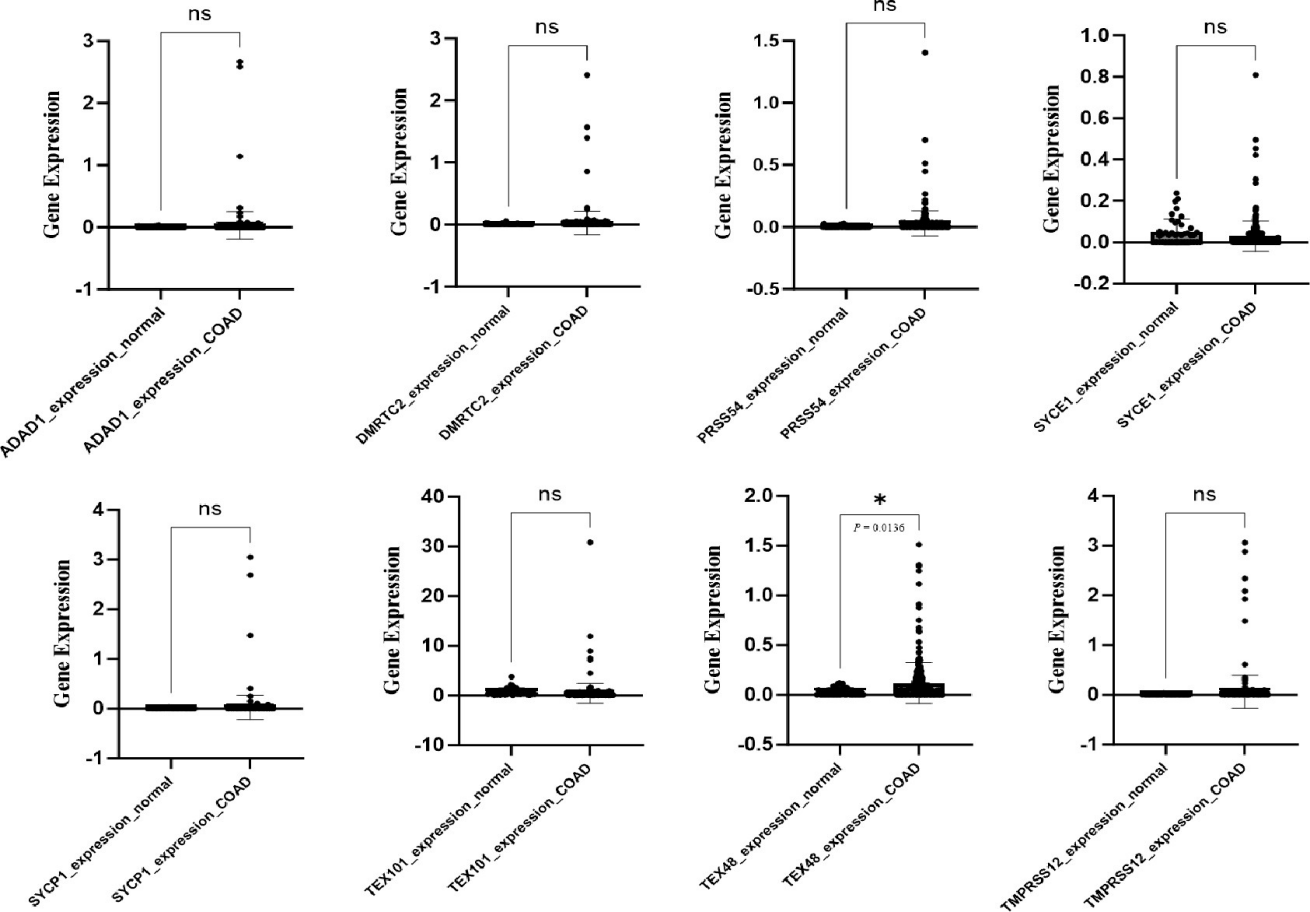
The expression levels of *ADAD1*, *DMRTC2*, *PRSS54*, *SYCE1*, *SYCP1*, *TEX101*, *TEX48*, and *TMPRSS12* in the NC and CC tissue samples from the TCGA-COAD dataset. The box plots of the CT genes show the differences in gene expression levels between the 41 NC and 308 CC tissue samples. *p ≤ 0.05. Abbreviations: COAD: Colon adenocarcinoma; NC: Normal colon; ns: Not-significant.

**Fig 3:**
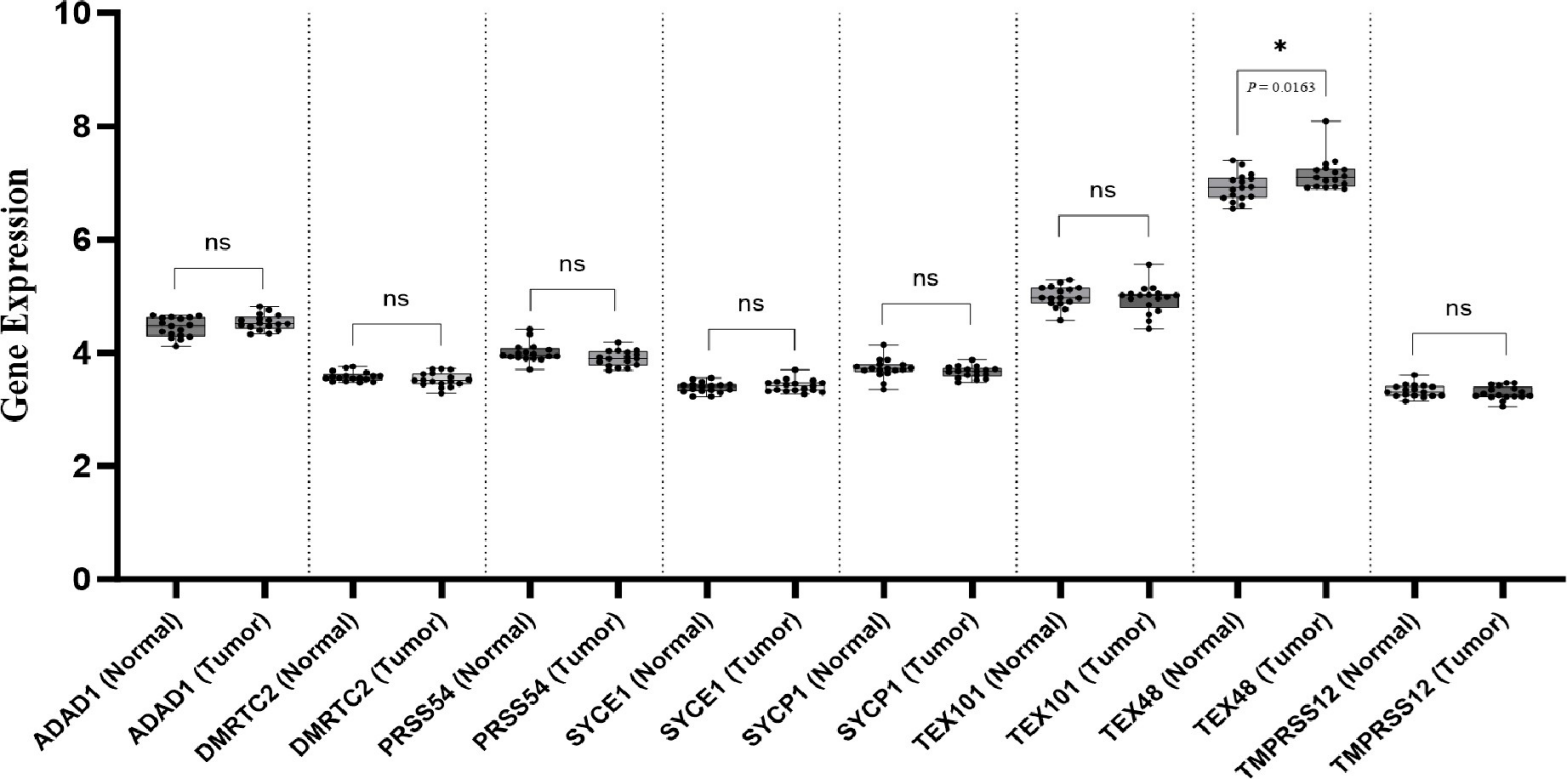
The expression levels of *ADAD1*, *DMRTC2*, *PRSS54*, *SYCE1*, *SYCP1*, *TEX101*, *TEX48*, and *TMPRSS12* in the NC and CC tissue samples from the GEO-GSE32323 database. The box plots of the CT genes show the differences in gene expression levels between the 17 NC and 17 CC tissue samples. *p ≤ 0.05. Abbreviations: CC: Colon cancer; NC: Normal colon; ns: Not-significant.

We validated our findings in two cohorts, one from the Cancer Genome Atlas (TCGA) database (Fig 2) and the other from the GEO-GSE32323 dataset from the Gene Expression Omnibus (GEO) database (GEO-GSE32323; Fig 3). The first cohort consisted of 308 CC and 41 NC tissue samples. The RNA-seq data showed no significant differences in the expression levels of the *ADAD1*, *DMRTC2*, *PRSS54*, *SYCE1*, *SYCP1*, *TEX101*, and *TMPRSS12* genes between the CC and NC tissue samples. However, the *TEX48* gene expression level increased significantly in the CC samples compared with the NC samples (mean expression level: 0.121 vs. 0.028; *p* = 0.0136). The second validation cohort consisted of 17 CC and 17 matched NC tissues. As shown in Fig 3, no significant increases in the expression levels of the *ADAD1*, *DMRTC2*, *PRSS54*, *SYCE1*, *SYCP1*, *TEX101*, and *TMPRSS12* genes were confirmed in this cohort. However, a significant increase in the *TEX48* expression level was found in the CC tissue samples compared with the matched NC tissue samples (mean expression level: 7.157 vs. 6.926; p = 0.0163).

### 5-aza-CdR treatment enhanced the expression of testis-restricted genes in two CC cell lines

The RT-PCR analysis results of the CC and matching NC tissue samples demonstrated that the *ADAD1*, *DMRTC2*, *PRSS54*, *SYCE1*, *SYCP1*, *TEX101*, *TEX48*, and *TMPRSS12* genes showed testis-restricted expressions but no positive expression signals (Fig 1). Consequently, we designed this experiment to examine whether treatment with a hypomethylating modulator can induce the expression activities of these genes in CC. Thus, two CC cell lines, HCT116 and Caco-2, were exposed to 10-μM 5-aza-CdR (DNMTi) for 72 h. Another group of each CC cell line was treated with dimethyl sulfoxide (DMSO), to dissolve the 5-aza-CdR drug in order to compare the gene expression between the two groups.

**Fig 4:**
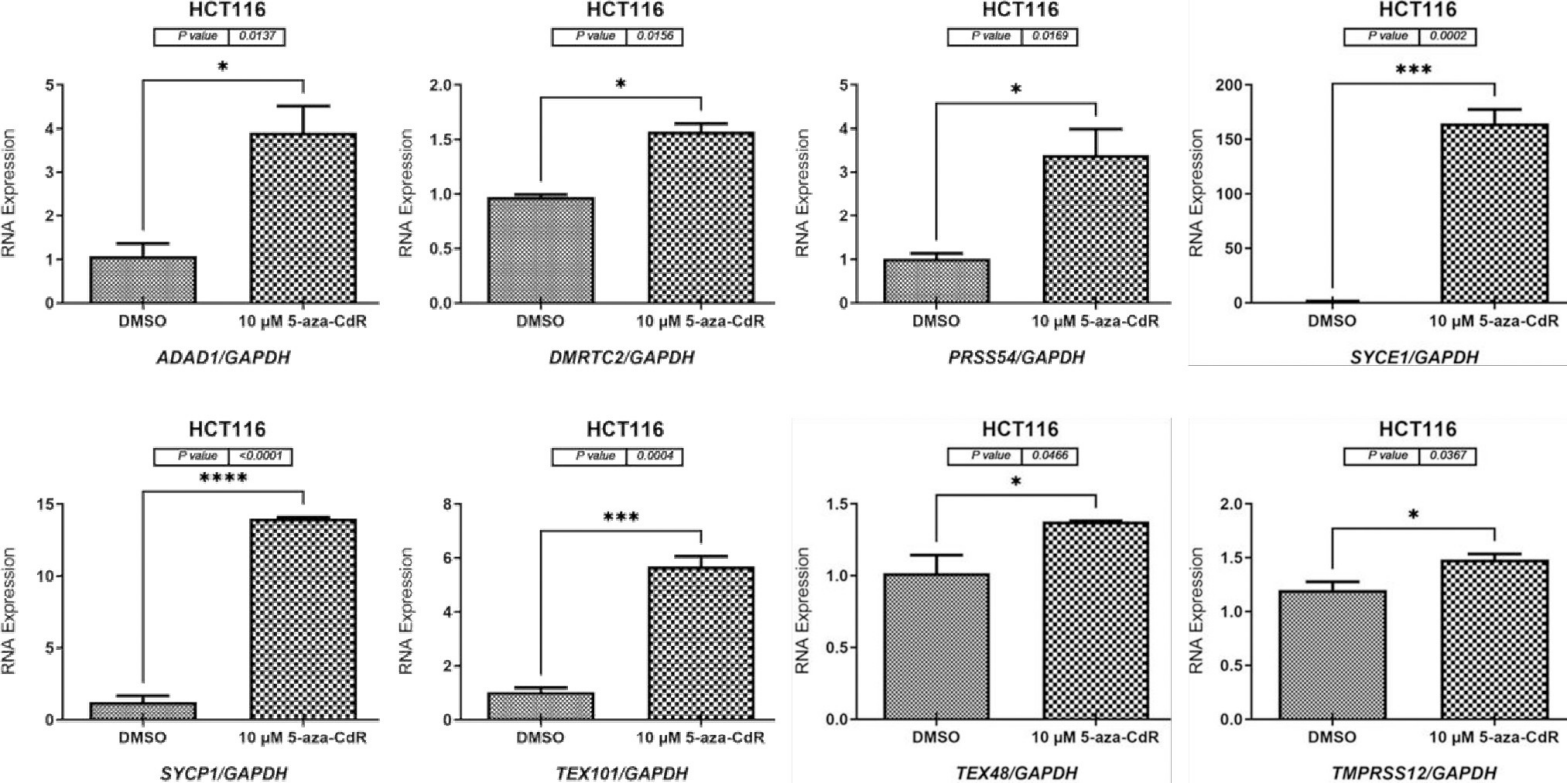
qRT-PCR analyses of the *ADAD1*, *DMRTC2*, *PRSS54*, *SYCE1*, *SYCP1*, *TEX101*, *TEX48*, and *TMPRSS12* gene expressions in the HCT116 cell line treated with 5-aza-CdR. The gene expression levels of *ADAD1*, *DMRTC2*, *PRSS54*, *SYCE1*, *SYCP1*, *TEX101*, *TEX48*, and *TMPRSS12* in the HCT116 cell line treated with 10 μM of 5-aza-CdR medication for 72 h are shown graphically as bar charts. As DMSO was used to dissolve the 5-aza-CdR solution, this was the treatment given to the HCT116 cells in the control group. To normalize the expression data, the mRNA expression level of the *GAPDH* housekeeping gene was used. Each gene has three independent qRT-PCR replicates, and the error bars show the standard error of the mean of the results. Statistical significance was assumed for all *p* values (*p ≤ 0.05, **p ≤ 0.01, ***p ≤ 0.001, ****p ≤ 0.0001).

**Fig 5:**
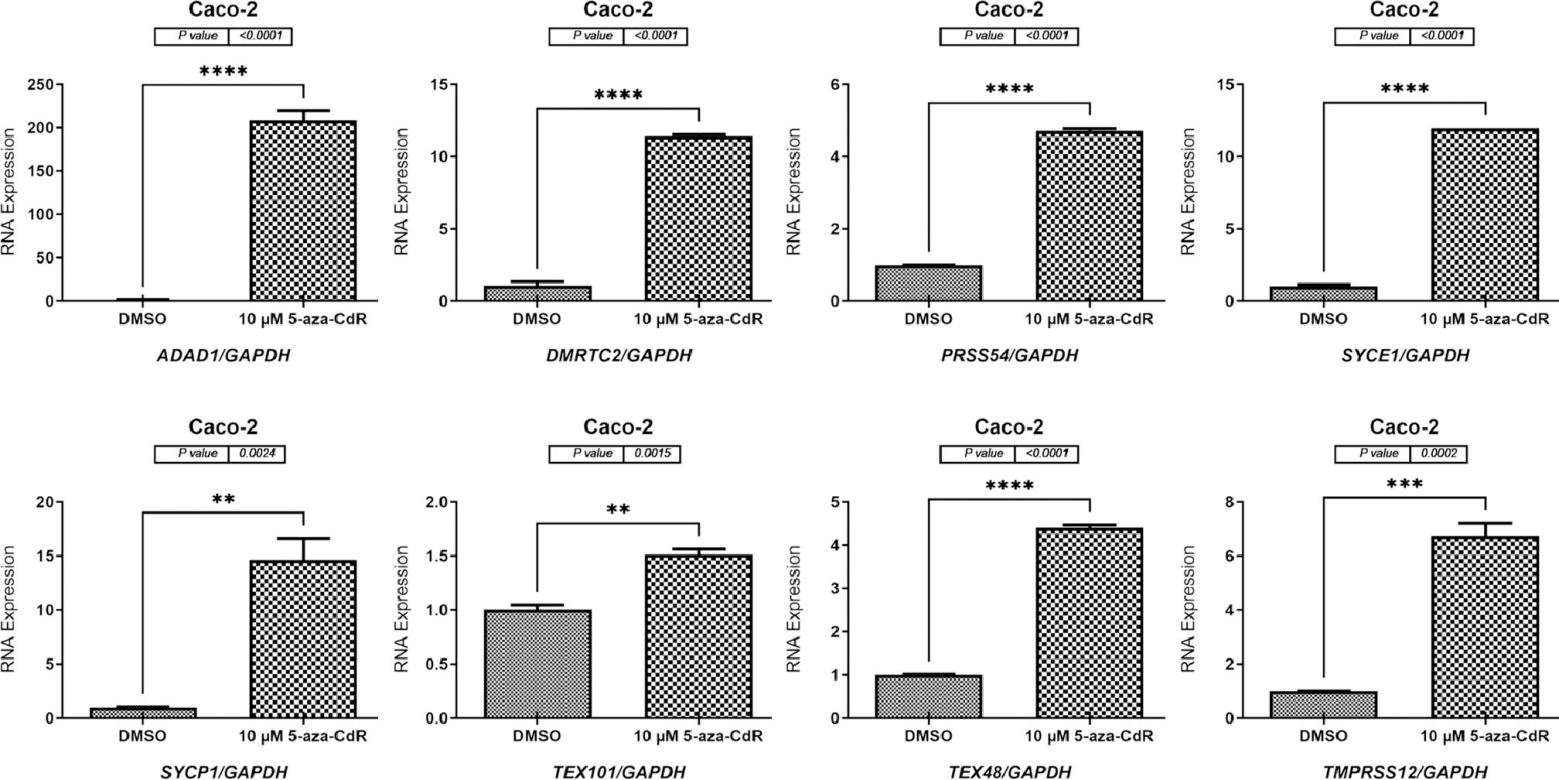
qRT-PCR analyses of the *ADAD1*, *DMRTC2*, *PRSS54*, *SYCE1*, *SYCP1*, *TEX101*, *TEX48*, and *TMPRSS12* gene expressions in the Caco-2 cell line treated with 5-aza-CdR. The gene expression levels of *ADAD1*, *DMRTC2*, *PRSS54*, *SYCE1*, *SYCP1*, *TEX101*, *TEX48*, and *TMPRSS12* in the Caco-2 cell line treated with 10 μM of 5-aza-CdR medication for 72 h are shown graphically as bar charts. As DMSO was used to dissolve the 5-aza-CdR solution, this was the treatment given to the Caco-2 cells in the control group. To normalize the expression data, the mRNA expression level of the *GAPDH* housekeeping gene was used. Each gene has three independent qRT-PCR replicates, and the error bars show the standard error of the mean of the results. Statistical significance was assumed for all *p* values (*p ≤ 0.05, ** *p*≤ 0.01, *** *p*≤ 0.001, ****p ≤ 0.0001).

Using qRT-PCR, the *ADAD1*, *DMRTC2*, *PRSS54*, *SYCE1*, *SYCP1*, *TEX101*, *TEX48*, and *TMPRSS12* gene expression levels were significantly higher by 4-fold (*p* = 0.0137), 1.5-fold (*p* = 0.0156), 3.5-fold (*p* = 0.0169), 150-fold (*p* = 0.0002), 14-fold (*p* ≤ 0.0001), 7-fold (*p* = 0.0004), 1.4-fold (*p* = 0.0466), and 1.5-fold (*p* = 0.0367), respectively, in the 5-aza-CdR-treated HCT116 cells than in the DMSO-treated HCT116 cells (Fig 4). Consistently, DNMTi (5-aza-CdR) treatment of the Caco-2 cells significantly increased the expression levels of the *ADAD1*, *DMRTC2*, *PRSS54*, *SYCE1*, *SYCP1*, *TEX101*, *TEX48*, and *TMPRSS12* genes by 200-fold (*p* ≤ 0.0001), 11-fold (*p* ≤ 0.0001), 4-fold (*p* ≤ 0.0001), 11-fold (*p* ≤ 0.0001), 14-fold (*p* = 0.0024), 1.5-fold (*p* = 0.0015), 4-fold (*p* ≤ 0.0001), and 6-fold (*p* = 0.0002), respectively, in the 5-aza-CdR-treated group compared to DMSO-treated cells (Fig 5).

### TSA treatment increased the expression of testis-restricted genes in two CC cell lines

HCT116 and Caco-2 cells were treated with the HDACi agent (TSA) to determine whether the histone deacetylation mechanism plays a role in silencing the testis-restricted genes, namely *ADAD1*, *DMRTC2*, *PRSS54*, *SYCE1*, *SYCP1*, *TEX101*, *TEX48*, and *TMPRSS12*, which were not expressed in the CC tissue samples from the Saudi patients. The dosage of the TSA agent was 100 nM for a period of 48 h, and DMSO was used for the treatment of both CC cell lines for the same period to dissolve the TSA drug in order to compare the gene expression between the two groups.

**Fig 6:**
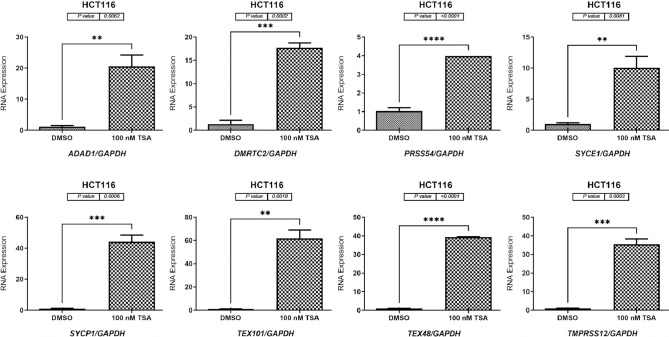
qRT-PCR analyses of *ADAD1*, *DMRTC2*, *PRSS54*, *SYCE1*, *SYCP1*, *TEX101*, *TEX48*, and *TMPRSS12* in the HCT116 cell line treated with TSA. The gene expression levels of *ADAD1*, *DMRTC2*, *PRSS54*, *SYCE1*, *SYCP1*, *TEX101*, *TEX48*, and *TMPRSS12* in the HCT116 cell line treated with 100 nM TSA for 48 h are shown graphically as bar charts. As DMSO was used to dissolve the 5-aza-CdR solution, this was the treatment given to the HCT116 cells in the control group. To normalize the expression data, the mRNA expression level of the *GAPDH* housekeeping gene was used. Each gene has three independent qRT-PCR replicates, and the error bars show the standard error of the mean of the results. Statistical significance was assumed for all *p* values (*p ≤ 0.05, **p ≤ 0.01, ***p ≤ 0.001, and ****p ≤ 0.0001).

**Fig 7:**
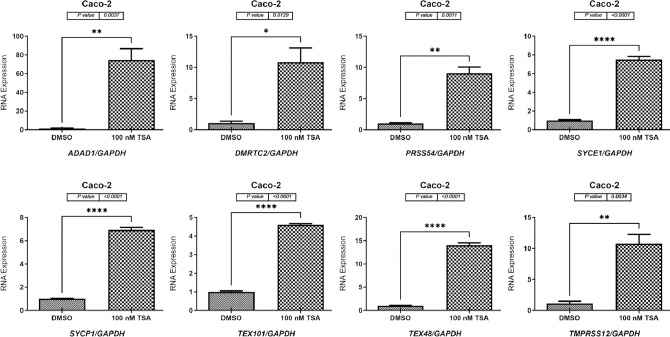
qRT-PCR analyses of *ADAD1*, *DMRTC2*, *PRSS54*, *SYCE1*, *SYCP1*, *TEX101*, *TEX48*, and *TMPRSS12* in the Caco-2 cell line treated with TSA. The gene expression levels of *ADAD1*, *DMRTC2*, *PRSS54*, *SYCE1*, *SYCP1*, *TEX101*, *TEX48*, and *TMPRSS12* in the Caco-2 cell line treated with 100 nM TSA for 48 h are shown graphically as bar charts. As DMSO was used to dissolve the 5-aza-CdR solution, this was the treatment given to the Caco-2 cells in the control group. To normalize the expression data, the mRNA expression level of the *GAPDH* housekeeping gene was used. Each gene has three independent qRT-PCR replicates, and the error bars show the standard error of the mean of the results. Statistical significance was assumed for all *p* values (*p ≤ 0.05, **p ≤ 0.01, ***p ≤ 0.001, and ****p ≤ 0.0001).

For the HCT116 cells, the qRT-PCR results showed that the *ADAD1*, *DMRTC2*, *PRSS54*, *SYCE1*, *SYCP1*, *TEX101*, *TEX48*, and *TMPRSS12* genes were significantly increased by 20-fold (*p* = 0.0062), 17-fold (*p* = 0.0002), 4-fold (*p* ≤ 0.0001), 10-fold (*p* = 0.0081), 40-fold (*p* = 0.0006), 60-fold (*p* = 0.0010), 40-fold (*p* < 0.0001), and 35-fold (*p* = 0.0003), respectively, in the TSA-treated cells compared to DMSO-treated group (Fig 6). Consistently, TSA treatment of the Caco-2 cells significantly increased the expression levels of the *ADAD1*, *DMRTC2*, *PRSS54*, *SYCE1*, *SYCP1*, *TEX101*, *TEX48*, and *TMPRSS12* genes by 70-fold (*p* = 0.0037), 10-fold (*p* = 0.0129), 9-fold (*p* = 0.0011), 7.5-fold (*p* < 0.0001), 7-fold (*p* < 0.0001), 4.5-fold (*p* < 0.0001), 14-fold (*p* < 0.0001), and 10-fold (*p* = 0.0034), respectively, in the TSA-treated Caco-2 cells compared to DMSO-treated cells (Fig 7).

## Discussion

There is an urgent need for better techniques to identify and treat CC at an early stage in Saudi Arabia, as it is often diagnosed at advanced stages and mortality from this type of cancer is increasing. This is consistent with global trends, where the majority of cancer-related deaths and illnesses are attributed to late-stage diagnoses. It is therefore possible to improve diagnostic accuracy and treatment effectiveness by exploring new biomarkers of CC, such as CT genes. Therefore, the goal of this research was to detect *ADAD1*, *DMRTC2*, *PRSS54*, *SYCE1*, *SYCP1*, *TEX101*, *TEX48*, and *TMPRSS12* expressions in human CC tissue samples compared with neighboring NC tissue samples and to evaluate whether these genes have potential as biomarkers for the early detection of CC. In the RT-PCR validation research, all gene expressions were determined to be specific to the testis and undetectable in the CC tissue samples. Given that their expression patterns were consistent with those in the normal testis, they were designated as testis-specific genes. The lack of detection of these genes in patients with CC may be due to the insufficient sample size. Previous research [35,36], has linked CT genes to many types of cancer, such as CC. This means that these genes can potentially be used as indications for diagnosis or prognosis. Unfortunately, the understanding of CT gene expression patterns in CC tissues is currently insufficient, especially concerning the Saudi population. To address this knowledge gap, we analyzed the expression patterns of a specific group of CT genes (*ADAD1*, *DMRTC2*, *PRSS54*, *SYCE1*, *SYCP1*, *TEX101*, *TEX48*, and *TMPRSS12*) in CC tissues. We used rigorous methods in primer design and experimental techniques to ensure the accuracy and reliability of our results. To minimize the risk of false-positive results due to genomic DNA contamination, primers were carefully selected from distinct exons of each gene. Table 2 shows the successful validation of the expression of the eight genes in CC tissue samples using appropriate primers in our RT-PCR studies. These investigations were performed on fresh tissue samples obtained from 15 CC patients and matched individuals with NC.

There were variations in gene expression patterns compared to previous researches [37,38], particularly in CT gene expression in CC tissues. The results of these studies revealed significant upregulation of CT genes in CC tissue samples obtained from Saudi patients. This is in stark contrast to previous studies [11,39–41], which reported minimal to no expression of these genes in CC. Differences in study populations, methods or sample processing techniques may explain this discrepancy. Conducting research targeting specific populations is essential to the development of cancer biomarkers. Therefore, further testing in additional CC tissue samples and tissue samples from other multiple malignancies not covered in this study may show positive expressions of these genes; hence, these genes were not excluded as possible cancer markers. *Prss54* expression is confined to the testes in both mice and humans, as shown by the RT-PCR analysis of a panel of tissue cDNAs from both species, as described in a previous work [42]. Previous research has also shown that *TEX101* expression was absent in all breast cancer specimens and cell lines tested [43], whereas *TEX101* overexpression was observed in head and neck carcinoma [44] and chronic myeloid leukemia [45]. These recent findings shed light on CT gene expression patterns in CC. Additionally, using primers derived from many exons increases the reliability of our results and reduces the risk of misinterpretations. The study highlights the importance of using genetic indicators in clinical settings to improve patient outcomes. Furthermore, this adds to the growing body of evidence indicating that CT genes play a role in the development of CC. Further research is needed to better understand how CT gene expression contributes to the development of CC, its implications for diagnosis, and the potential for targeted therapy in the Saudi population.

In addition, this study included two validation cohorts (TCGA and GEO databases) and used the qRT-PCR test for the gene expression analysis as per the method of earlier researchers. In CC and NC tissue samples, the data from the cohorts demonstrated that all CT genes were expressed at low levels. Importantly, in both cohorts, *TEX48* expression was much higher in CC tissues than in NC tissues. TEX48 is a gene on chromosome 9q32 that is exclusive to the testis, although its exact function in healthy and diseased tissues remains unknown [46]. Results from previous studies highlight the involvement of TEX48 in spermatogenesis and its significant expression in the testes [47]. Unfortunately, research on its role in cancer biology is limited. Further investigation of the role of TEX48 in carcinogenesis is warranted in light of our results, which suggest a possible correlation between *TEX48* expression and CC. There may be population-specific changes or variations in the pathophysiology of CC, as indicated by the fact that *TEX48* is upregulated in CC tissue samples but not expressed in the Saudi cohort [47]. Further research is needed on the TEX48 gene as it is highly expressed in the validation cohort but not in the Saudi cohort. Determining the functional role of TEX48 in CC growth and development should be the focus of future studies. Studying the molecular pathways responsible for TEX48 dysregulation in CC may also lead to new treatment options for this cancer [48]. These findings highlight the need for studies based on specific populations when trying to identify biomarkers, and they add to the growing body of evidence that CT genes play a role in cancer development. To confirm our results and clarify the therapeutic significance of *TEX48* expression in CC, further research is needed as per the exploration of earlier researcher [46].

Tumorigenesis has been shown to have essential epigenetic drivers. Currently, the biological roles of CT genes remain to be clarified. New findings have shown that they may play a role in carcinogenesis by controlling transcriptional activity [49]. The epigenetic control of gene expression, particularly the reduction of histone deacetylation and DNA methyltransferase, was also investigated for the following genes: *ADAD1*, *DMRTC2*, *PRSS54*, *SYCE1*, *SYCP1*, *TEX101*, *TEX48*, and *TMPRSS12*. Early-passage HCT116 and Caco-2 cell lines were exposed to either 5-aza-CdR (10 μM for 72 h) or TSA (100 nM for 48 h) after isolation from human CC tissues. The 5-aza-CdR drug treatment demonstrated that the expression levels of all tested genes were increased after 5-aza-CdR treatment in both CC cell types. The results of the 5-aza-CdR treatment indicated that similar treatments might activate the expressions of the examined genes but at different expression levels, as shown in Figs 4 and 5, which suggests the existence of gene specificity. This confirms previous the research finding that different CT genes were expressed at varying levels in 5-aza-CdR-treated CC cells [7,13,24]. This study finding raises the important issue of why induction was detected at such a high level in the CC cell lines after 5-aza-CdR treatment but not in the other cell lines after DMSO treatment. Researchers have observed that 5-aza-CdR treatment reduces the expression level of the key enzyme involved in DNA methylation, DNA methyl-transferase 1 (*DNMT1*) [13]. Although 5-aza-CdR therapy has been shown to reduce the expression levels of certain *DNMT1* genes, additional studies are required to determine whether this holds true for all *DNMT* genes.

*TEX101* overexpression in individuals with chronic myeloid leukemia may be attributable to the hypomethylation of this gene. Many forms of cancer may benefit from a treatment strategy that combines epigenetic modulatory medicines with CT antigen immunotherapy [50]. In addition, the widespread tumor-specific expression of *TEX101* suggests that it may play a part in the molecular process of carcinogenesis or may just be a byproduct of hypomethylation in cancer. The associations between *TEX101* expression and tumor grade and stage are an important area for further research. Increasing *TEX101* expression levels using epigenetic techniques is a potential therapeutic approach. The limited expression of *TEX101* in the testis makes it both protective against metastasis and effective in eliciting an immune response. Despite the blood-testis barrier, it is nevertheless possible to trigger an immune response by expressing testis-specific genes elsewhere in the body. It may become a more viable target for immunotherapy if further research demonstrates protein-level TEX101 expression and the development of humoral responses against this gene in malignancies [45].

In contrast to DMSO treatment, TSA administration effectively inhibited histone deacetylation in both cell types examined, leading to increased expression levels of all eight genes examined. To control the activity of the genes studied, our research emphasizes the importance of inhibiting histone deacetylation. These genes play roles in several stages of tumor development, and epigenetic evidence suggests that there is a complex regulatory network that controls their activity. Several studies have shown the importance of CT genes in the origin and development of cancer [6,51]. These genes are associated with the spread of tumors, infiltration of cancer cells into surrounding tissues, spread of cancer to other parts of the body, and atypical expression of genes in cancer cells. Therefore, it is worth considering whether there is potential to improve the efficacy of alternative therapeutic approaches and reduce the cancer burden resulting from rapid growth by focusing on the regulation of CT gene expression.

Targeting histone deacetylation may have therapeutic implications in cancer treatment, as demonstrated by the upregulation of CT genes after TSA treatment. HDACIs, such as TSA, can regulate gene expression and restore proper epigenetic patterns, making them attractive as anticancer drugs [52]. HDACIs have the potential to inhibit tumor growth and metastasis while increasing the sensitivity of cancer cells to other therapies [53]. They achieve this by correcting aberrant epigenetic changes associated with cancer. Understanding the regulatory mechanisms that control CT genes is important because of the complex interplay between epigenetic modifications and gene expression. The next study is expected to focus on identifying new therapeutic targets and understanding the complex epigenetic landscape of CT genes in cancer cells. Studying the potential synergistic effects of HDACIs with other treatment techniques such as immunotherapy or chemotherapy could lead to the development of more potent combinatorial drugs for cancer treatment. The study results demonstrate the importance of histone deacetylation in regulating CT gene expression, suggesting that targeting these genes could be a viable therapeutic strategy for cancer treatment.

The current study highlights the need for careful interpretation due to the important factors associated with the selection of biopsy specimens from patients diagnosed with CC. It is important for us to recognize and address the limitations of our experimental methods, although our research provides valuable understanding of the likely biochemical pathways involved in the development of CC. Biopsy specimens have limitations when used for gene expression studies. Tumor tissues have inherent heterogeneity, which introduces the possibility of variation in sample quality. Biopsy specimens may not satisfactorily represent the complex nature of CC biology compared with specimens obtained after surgical excision, even with attempts to ensure specimen integrity. Previous research has highlighted problems with biopsy specimens, including the risk of sampling bias and inadequate representation of tumor heterogeneity.

In addition, the lack of clinical data or sufficient tissue samples in our investigation precluded the use of other experimental methods to confirm our results. The present investigation used biopsy specimens; Despite this, larger and potentially higher samples obtained after surgery are generally considered more accurate. To improve the reliability of study results and minimize possible sample bias, previous research has shown the need to use surgical tissues for genetic analysis (3, 4). Furthermore, it is important to be cautious when extrapolating our results to other populations. This caution is justified by the fact that our investigation exclusively used CC specimens and was only performed retrospectively within the borders of Saudi Arabia. Future research requires larger, multicenter cohorts encompassing different demographic and clinical settings to validate and apply our findings more broadly.

Although our research found that CC cell lines showed high expression of the eight genes mentioned above when treated with inhibitors of DNA methylation and histone deacetylation, we could not confirm these results at the protein level. Therefore, the implications of these findings for translation remain unclear. To obtain a more complete understanding of the molecular changes associated with CC, it is advisable in future research to validate changes in gene expression at the protein level using techniques such as Western blotting or immunohistochemistry. This research contributes to the understanding of the etiology of CC, it is imperative that we recognize and correct problems with our experimental methodology. To improve our understanding of CC biology and identify potential therapeutic targets, it is crucial to overcome these limitations by conducting larger prospective studies involving different populations and rigorously validating the results at the protein level.

## Conclusions

The study results showed that the expressions of the *ADAD1*, *DMRTC2*, *PRSS54*, *SYCE1*, *SYCP1*, *TEX101*, *TEX48*, and *TMPRSS12* genes were detected only in the testis tissue and were undetectable in either the CC or adjacent NC tissue samples. Therefore, more testing is needed to identify possible cancer marker candidates in CC tissues not examined here and tissues from diverse malignancies. Furthermore, the results of the *in vitro* experiment showed that the expression levels of the genes were increased when methylation and histone deacetylation were inhibited in the CC cell lines. The significant role of this epigenetic modulator in the transcriptional activation of the examined genes suggests that it may be included in future cancer immunotherapies. The long-term results of treatment with 5-aza-CdR alone or in conjunction with a TSA agent warrant further investigation.

## Supporting information

S1 Fig (PDF)

S1 Raw images (PDF)

S1 Data (XLS)

S2 Data (XLS)
