# Supplementary material for: Differential expression and regulation of ADAD1, DMRTC2, PRSS54, SYCE1, SYCP1, TEX101, TEX48, and TMPRSS12 gene profiles in colon cancer tissues and their in vitro response to epigenetic drugs
Source: PLoS One. 2024 Aug 29;19(8):e0307724. doi: 10.1371/journal.pone.0307724 (PMC11361649; doi:10.1371/journal.pone.0307724)
Supplement: S1 Fig — (PDF) [file pone.0307724.s001.pdf]

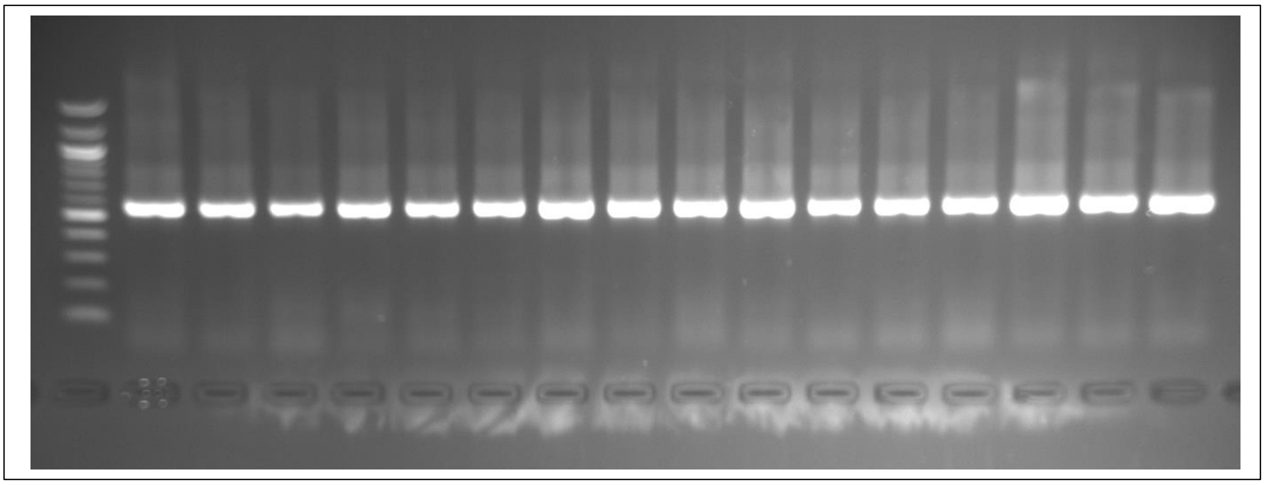

ACTB in NC samples

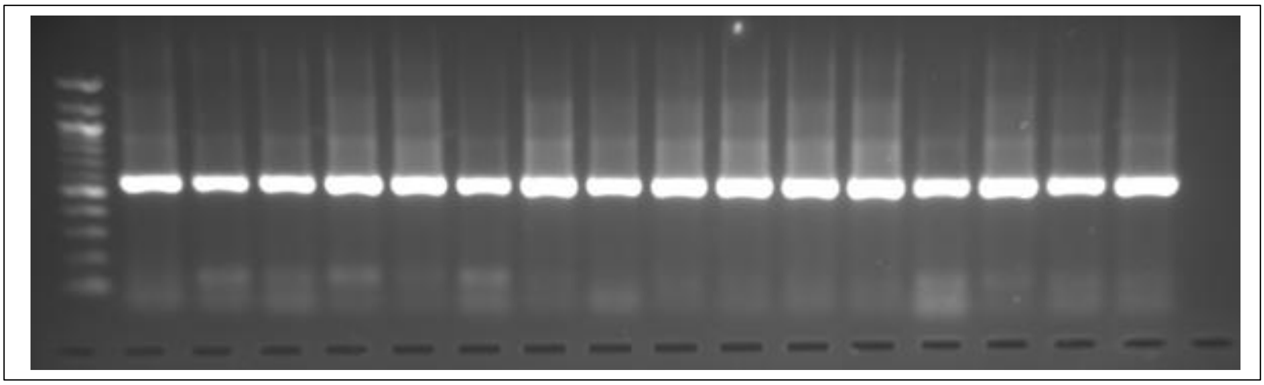

ACTB in CC samples

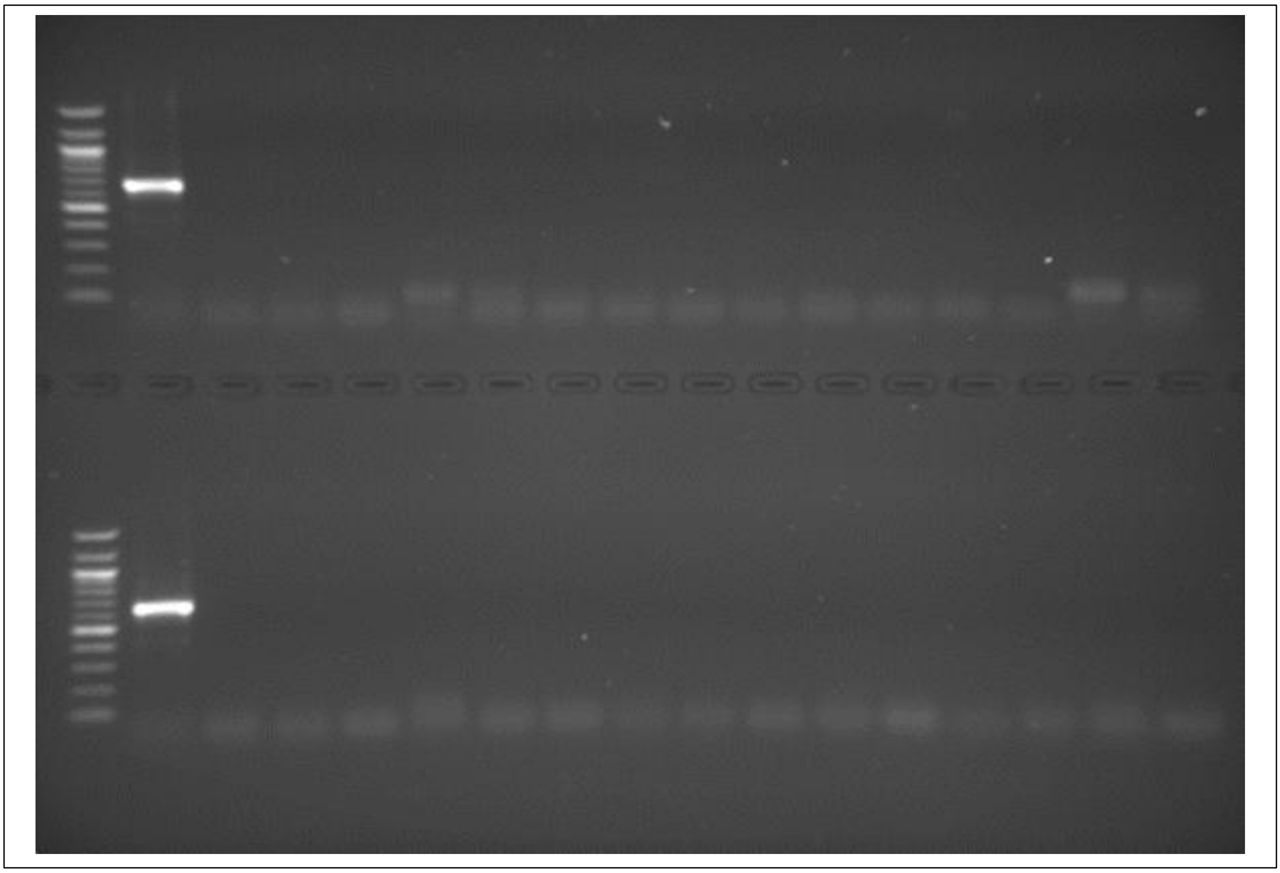

ADAD1 in NC samples up and CC samples down

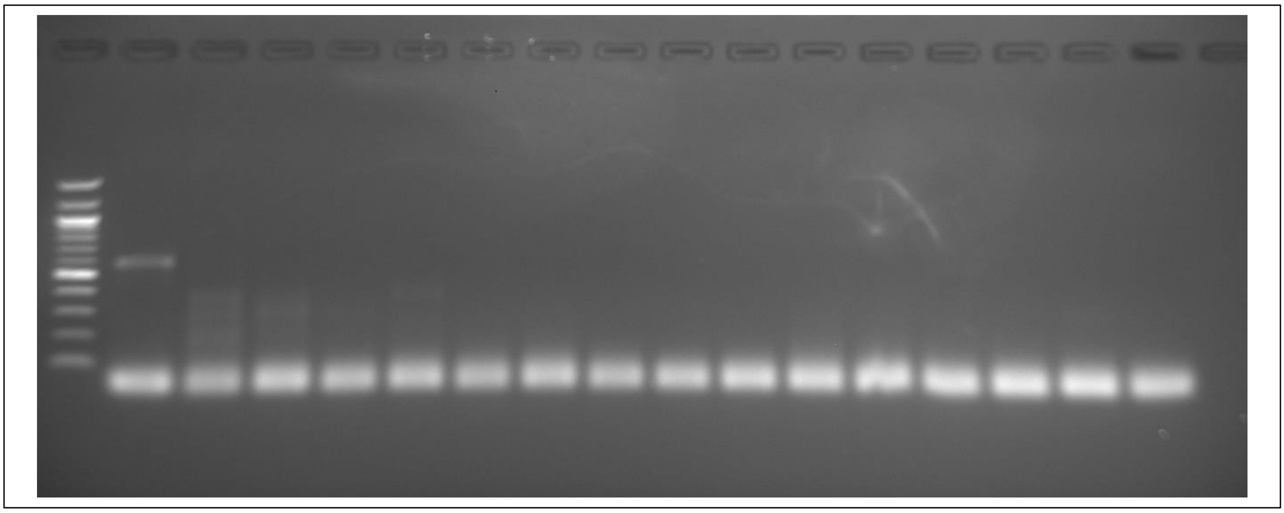

DMRTC2 in NC samples

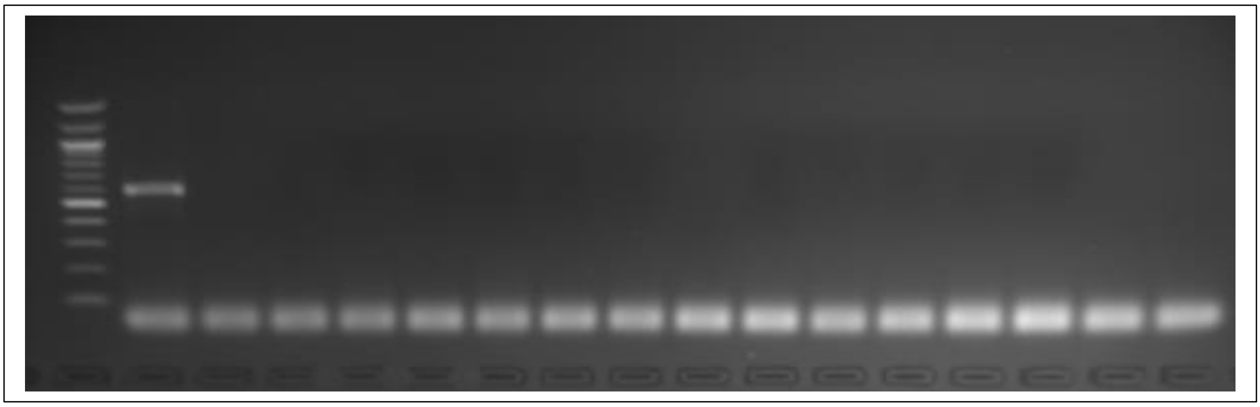

DMRTC2 in CC samples

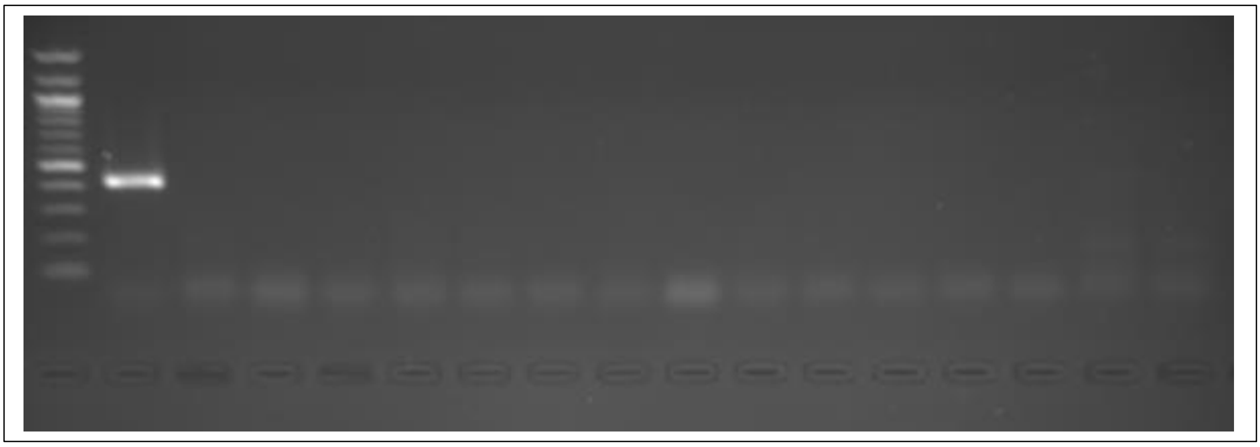

PRSS54 in NC samples

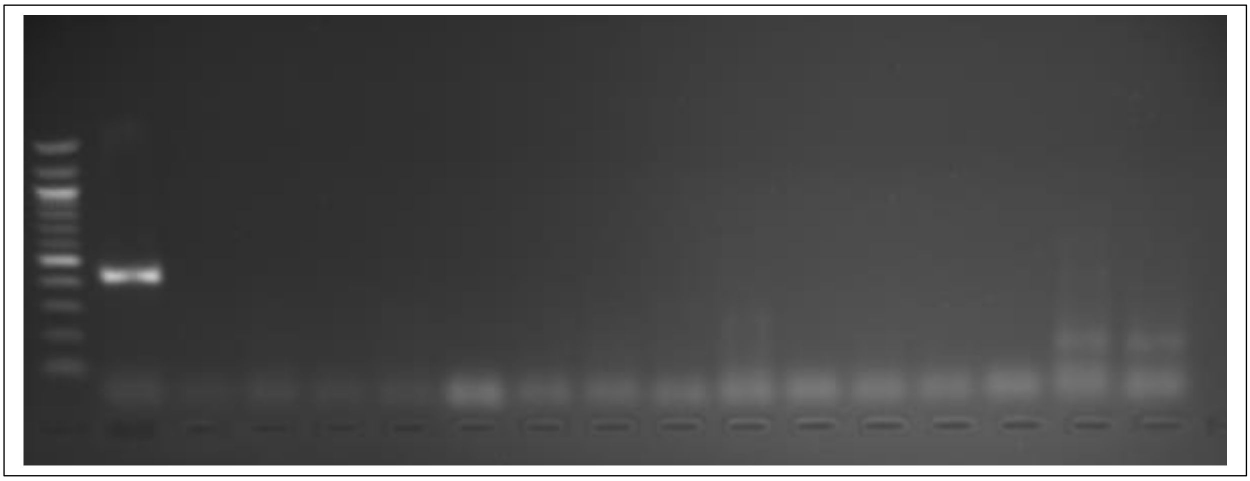

PRSS54 in CC samples

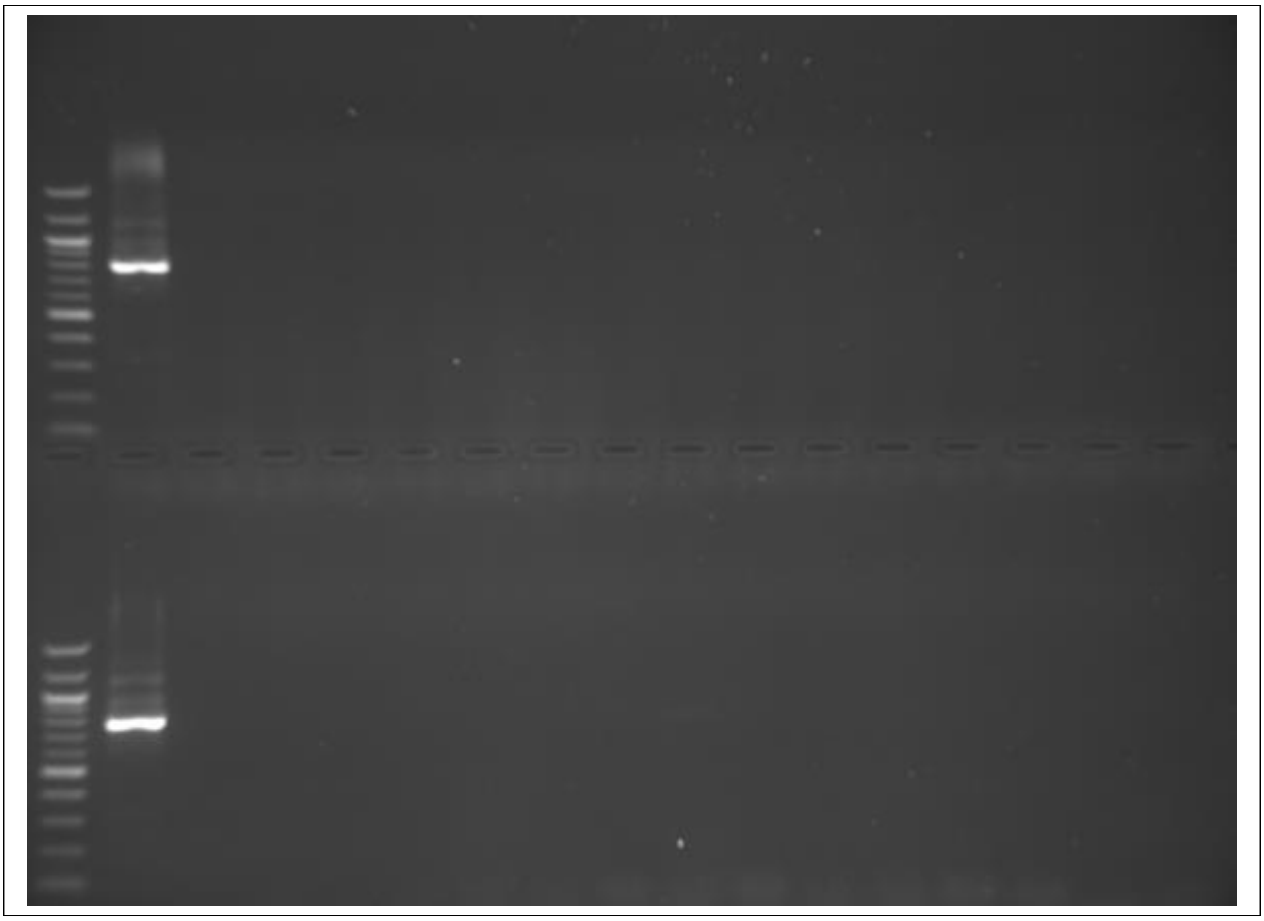

SYCE1 in CC samples up and NC samples down

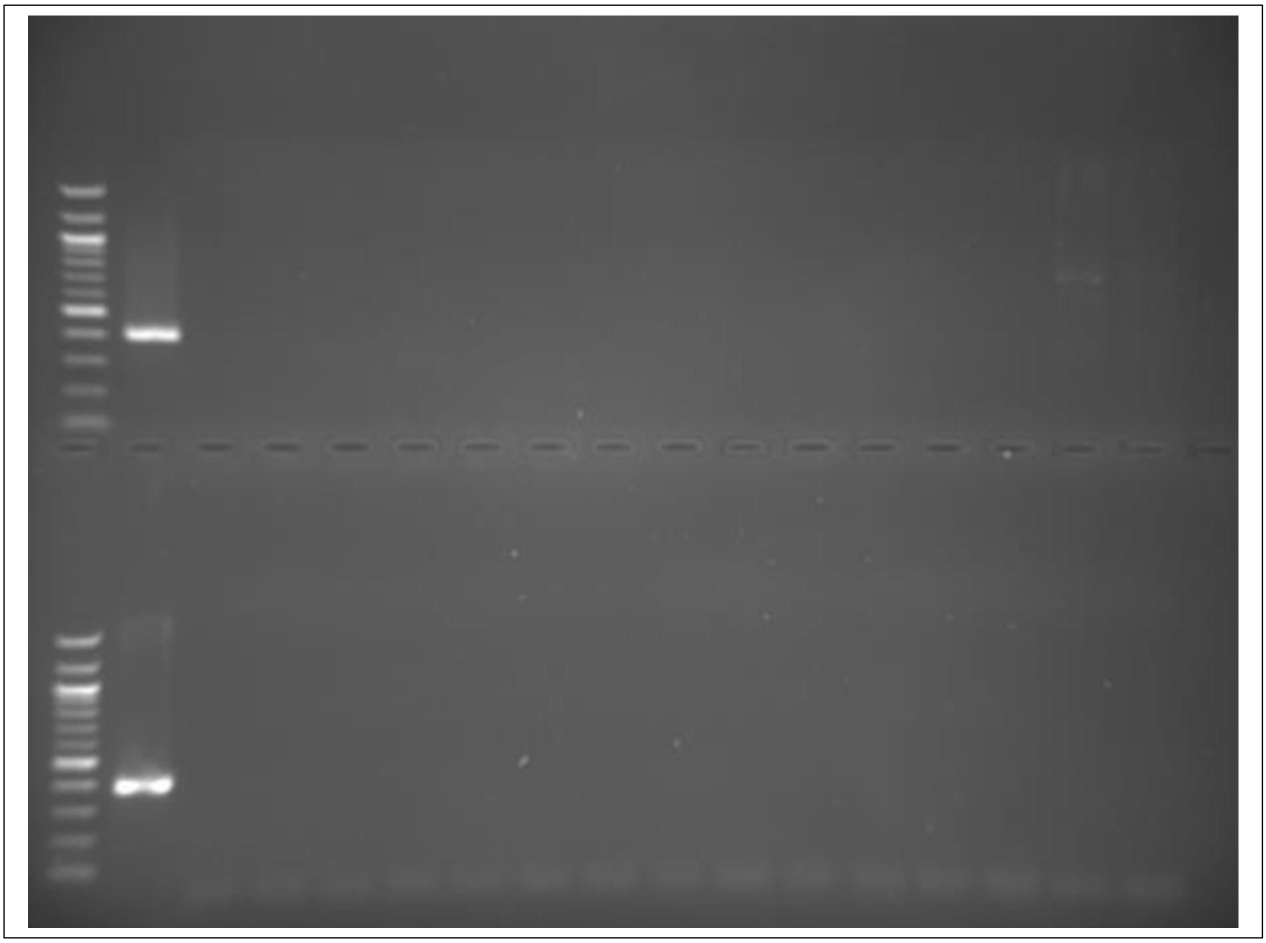

SYCP1 in CC samples up and NC samples down

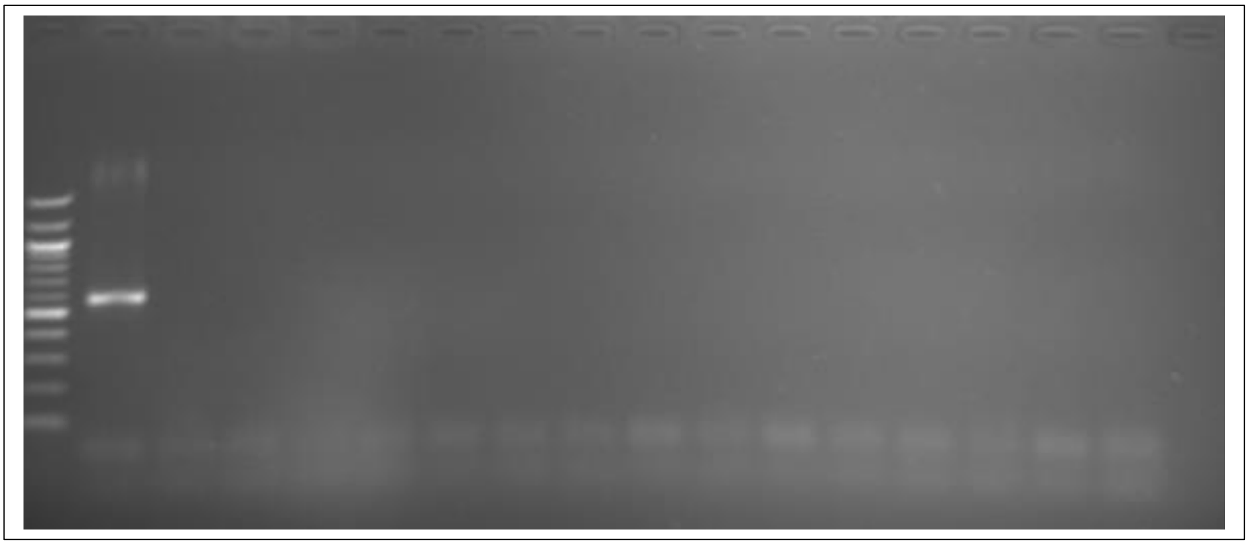

TEX101 in NC samples

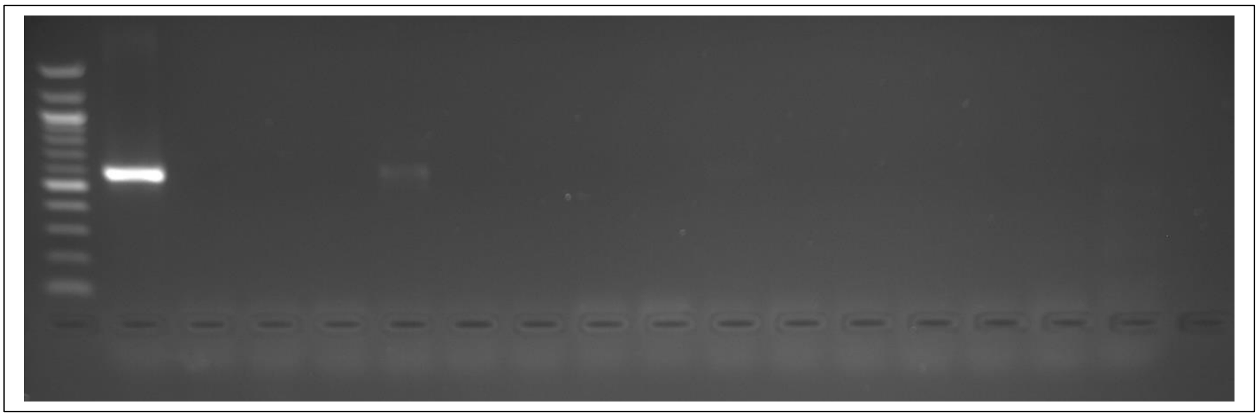

TEX101 in CC samples

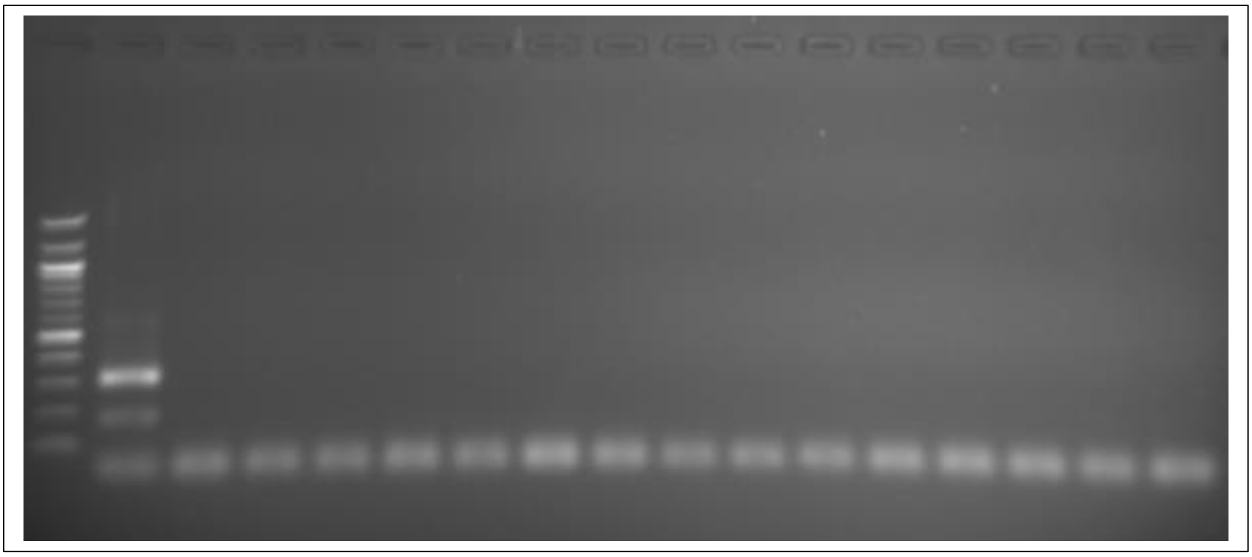

TEX48 in NC samples

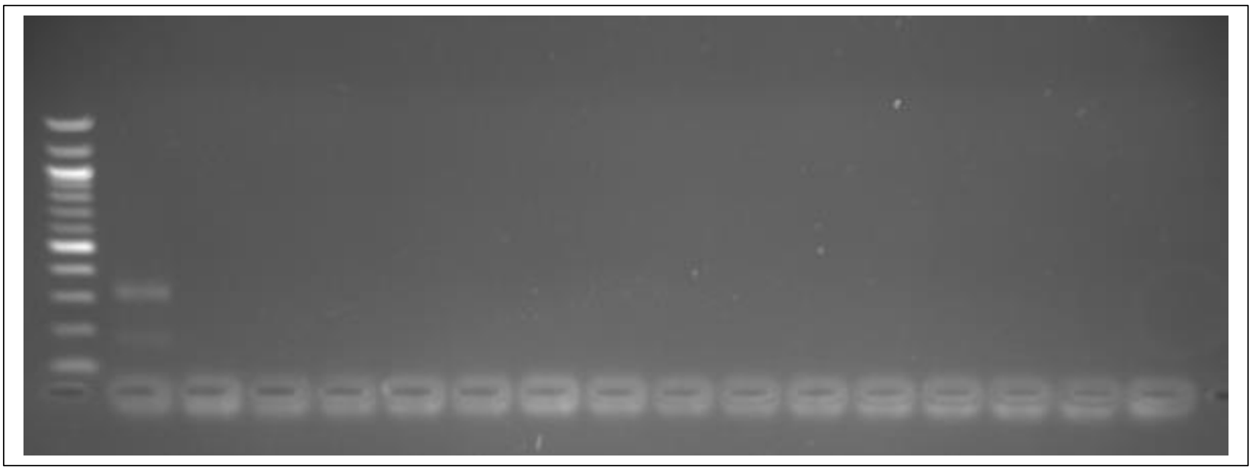

TEX48 in CC samples

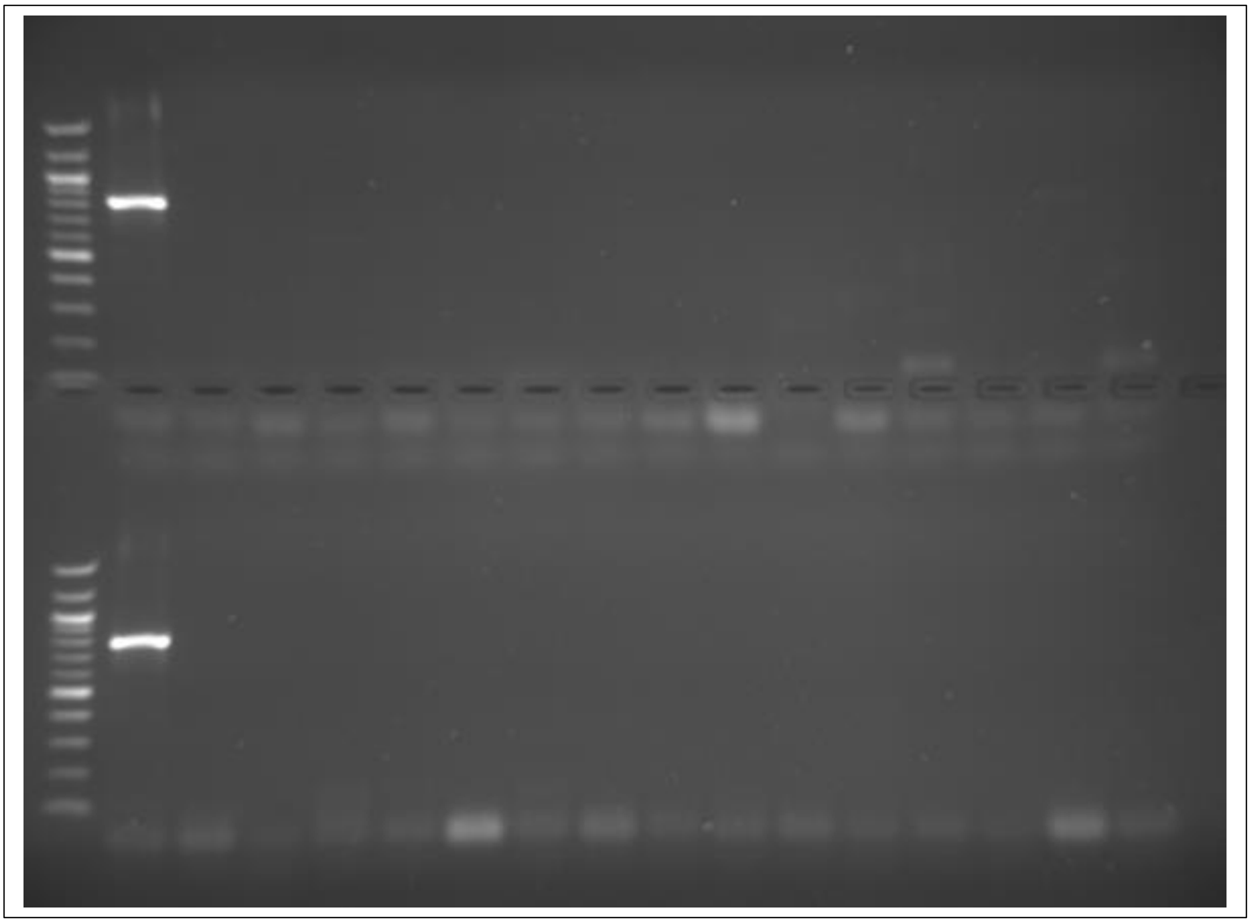

TMPRSS12 in CC samples up and NC samples down
